# Root traits correlate with crop rhizosphere microbiome diversity independent of legume relatedness

**DOI:** 10.1093/ismeco/ycag087

**Published:** 2026-04-14

**Authors:** Justin D Stewart, Malin Klein, Sébastien Jaupitre, Loreto Oyarte-Galvez, Lemeng Dong, Harro J Bouwmeester, E Toby Kiers, Vasilis Kokkoris, James T Weedon

**Affiliations:** Section of Ecology and Evolution, Amsterdam Institute for Life and Environment, Vrije Universiteit Amsterdam, Amsterdam 1081 HV, North Holland, The Netherlands; Section of Ecology and Evolution, Amsterdam Institute for Life and Environment, Vrije Universiteit Amsterdam, Amsterdam 1081 HV, North Holland, The Netherlands; Plant Hormone Biology, Swammerdam Institute for Life Sciences, University of Amsterdam, Amsterdam 1012 WX, North Holland, The Netherlands; Plant Hormone Biology, Swammerdam Institute for Life Sciences, University of Amsterdam, Amsterdam 1012 WX, North Holland, The Netherlands; Section of Ecology and Evolution, Amsterdam Institute for Life and Environment, Vrije Universiteit Amsterdam, Amsterdam 1081 HV, North Holland, The Netherlands; Physics of Behavior, AMOLF Institute, Amsterdam 1098 XG, North Holland, The Netherlands; Plant Hormone Biology, Swammerdam Institute for Life Sciences, University of Amsterdam, Amsterdam 1012 WX, North Holland, The Netherlands; Plant Hormone Biology, Swammerdam Institute for Life Sciences, University of Amsterdam, Amsterdam 1012 WX, North Holland, The Netherlands; Section of Ecology and Evolution, Amsterdam Institute for Life and Environment, Vrije Universiteit Amsterdam, Amsterdam 1081 HV, North Holland, The Netherlands; Section of Systems Ecology, Amsterdam Institute for Life and Environment, Vrije Universiteit Amsterdam, Amsterdam, 1081 HV, North Holland, The Netherlands; Section of Systems Ecology, Amsterdam Institute for Life and Environment, Vrije Universiteit Amsterdam, Amsterdam, 1081 HV, North Holland, The Netherlands

**Keywords:** rhizosphere, co-evolution, root traits, predictive model, domestication, phylogeny, machine learning, Bayesian

## Abstract

Predicting the composition of rhizosphere microbiomes has become increasingly important for sustainable agriculture. A key open question is whether a plant’s rhizosphere community is shaped more by the specific traits or host phylogeny, under different soil conditions. We conducted a greenhouse experiment on 15 legume species, including three pairs of crop-wild relative pairs, under different phosphorus conditions. We then sequenced the bacterial and fungal rhizosphere communities. Using Bayesian models, we found rhizosphere composition was shaped by individual species identity, independent of host phylogeny (intraclass correlation = 0.40–0.79). This suggests that closely related plants do not necessarily share similar rhizosphere microbiomes. These patterns remained consistent across host intraspecific variation and nutrient treatments. Using a custom-built root imaging platform, we quantified root architectural traits and applied machine learning to correlate with rhizosphere community composition (R^2^ = 0.46–0.80). Root diameter and carbon content were the strongest drivers. Notably, these key root traits were largely uncorrelated with phylogeny, yet strongly explained variation in rhizosphere community composition. Our results indicate that even closely related legume species may host divergent rhizosphere communities.

##  Introduction

Plants have formed and depended on symbiotic partnerships with microbes since their colonization of the terrestrial environment over 450 million years ago [1, 2]. Root microbiomes, including the rhizosphere surrounding plant roots enhance their host’s productivity, stress tolerance, and nutrient uptake [3, 4]. As interest grows in leveraging these rhizosphere communities for sustainable agriculture, the rhizosphere is increasingly considered part of the “next era of crop domestication” [5–7]. Harnessing the potential microbial communities holds great promise but relies on understanding how communities of microbes assemble on plant roots. The assembly of rhizosphere communities is a multi-step process influenced by multiple factors, and we lack a comprehensive understanding of how this process occurs [3, 8–11]. Understanding, and eventually predicting, the factors driving rhizosphere community assembly are critical for sustainable agriculture [8, 12–14].

One important factor driving rhizosphere community composition is the co-evolution of plants and microbes. While substantial progress has been made in identifying rhizosphere recruitment by plants, we still lack a clear understanding of what drives variation in whole rhizosphere community composition across closely related plant species [11, 15]. If certain microbial partners provide consistent fitness benefits, plants may favor their recruitment, potentially leading to microbial associations that are heritable [16, 17]. The co-evolution between hosts and their microbiomes is often evaluated using tests of phylogenetic signal, which assess whether closely related hosts harbor more similar microbial communities, than expected by chance [18–22]. Some studies suggest that inherited plant traits contribute to recruitment of specific rhizosphere communities, but not in all cases [14, 18, 23–26]. These mixed results a knowledge gap as to when host phylogeny shapes rhizosphere composition, especially as commonly used models often cannot disentangle effects of phylogeny, traits, and species identity. If host phylogeny reliably correlates with differences in rhizosphere community structure among plant hosts, this information could be combined with trait based and environmental variables. Together, these drivers may help identify relative similarities and differences in microbiome structure among closely related, unstudied plant species, with potential relevance for predicting microbiome compositions. Testing these multiple hypotheses is essential; without doing so, we risk misidentifying the true drivers of rhizosphere community composition.

Recent advances in statistical modeling, such as phylogenetic mixed-effects models, allow researchers to estimate both variance explained by phylogenetic signal, species identity independent of host phylogeny, and traits within the same model [27–29]. This represents a major advancement over previous approaches because it enables quantification of how strongly host phylogeny influences microbiome composition, in the context of other known drivers of variation. Such insights can guide microbiome breeding by clarifying how much efforts should rely on host phylogeny, or species-specific adaptations such as traits [26, 30–32]. These models also accommodate non-aggregated data, allowing the quantification of both inter- and intraspecific variation in microbiome composition and plant traits.

Research has recently focused on the role of plant traits such as root exudates in recruiting microbes to the rhizosphere [33]. In contrast, root architectural traits have received less attention, or have been limited to a handful of plant species [15, 34]. This is an oversight as roots are the plant organ that hosts the rhizosphere microbiome and is associated with nutrient uptake. Fine roots with high surface area enhance nutrient uptake, while coarse roots provide structural support but are less efficient in resource absorption [35, 36]. Such root architectural traits are highly plastic and responsive to environmental conditions, and traits such as root length, volume, and diameter are in some cases, but not always, correlated with rhizosphere community composition [26, 31, 32, 37, 38]. Root traits often vary widely within and across species, influenced by factors such as nutrient availability and even microbial interactions in the rhizosphere [39–41]. This variation complicates understanding how root traits contribute to community composition patterns, particularly across species growing under different environmental conditions.

Some root traits exhibit phylogenetic structure over deep evolutionary timescales (e.g. between families). In comparison less is known about how root trait variation and microbiome composition are linked within a single plant family, especially under different environmental conditions [42]. Still, globally important crops originate from just a few families (e.g. legumes), meaning understanding within family variation is an ideal target for study. Using appropriate models to test drivers of rhizosphere variation within plant families is required as closely related plant species can have exhibit highly divergent microbiomes and root traits, such as between domesticated crops and their wild relatives [32, 43–46]. Because many breeding programs focus on crops and their wild relatives, this represents a key gap in our understanding. To address these questions, experiments should include multiple plant species within the same family, grown in a common soil, with replicated individuals and detailed root phenotyping under contrasting phosphorus conditions. Legumes are ideal host plants because they include many globally cultivated crop and non-crop species, are found in all vegetated terrestrial ecosystems, and exhibit highly variable root architectures within a single plant family [47–50].

**Figure 1:**
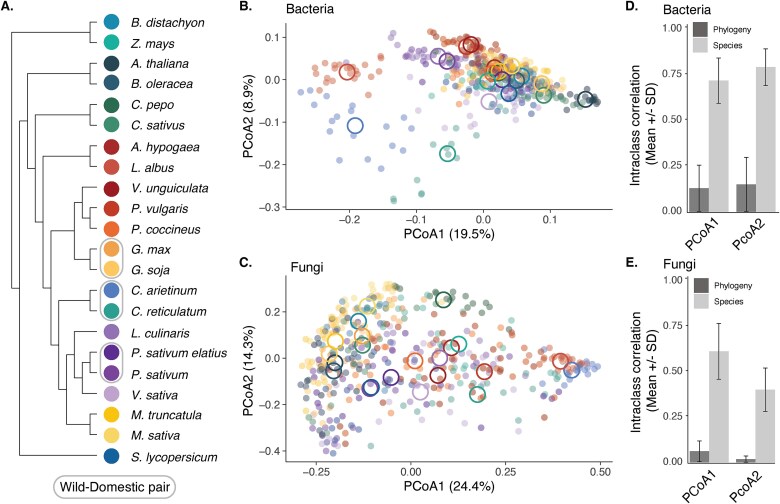
Community composition and phylogenetic signal of the rhizosphere microbiome. (A) Plants species in this study include 15 Fabaceae species and 7 outgroup species (two Cucurbitaceae, two Brassicaceae, one Solanaceae, two Poaceae), covering 135 million years of plant evolution. Plants were grown under two soil phosphorus treatment (high and low), and rhizosphere microbial communities were analyzed. PCoA was used to summarize microbial community composition for bacteria (B; PC1: 19.5%, PC2: 8.9%) and fungi (C; PC1: 24.2%, PC2: 14.3%). Small circles represent individual plants, and large circles indicate species-level means across replicates. Each circle is colored by plant species. To test if community composition was driven by host phylogeny or species effects independent of phylogeny, a series of Bayesian models were fitted and the ICC for each model random effect (species or phylogeny) was calculated. No single best model was selected and thus the mean and standard deviation across all models is shown. Species effects as opposed to host phylogeny dominated community composition for both bacteria (D) and fungi (E).

We addressed this by conducting a greenhouse experiment on 528 individual plants from 15 legume and 7 non-legume outgroup species spanning 135 million years of evolutionary history, including three pairs of crops and their wild relatives (Fig. 1A). Specifically we asked, (i) whether rhizosphere communities differ between different plant hosts, (ii) if so, is this variation better explained by host relatedness or root architectural traits, (iii) whether there are consistent relationships between root architectural traits and rhizosphere community composition across species, and (iv) whether domestication has similar effects on rhizosphere communities and root architectural traits across legumes. For this, plants were grown under two soil phosphorus conditions. Phosphorus is a key limiting nutrient in many terrestrial ecosystems and plays a central role in global agriculture, and influences both root architecture and soil microbiome composition [51]. Variation in phosphorus availability arises both from inherent differences in soil development and from inputs associated with fertilizer use in managed systems, making it an ecologically and agriculturally relevant [52]. After harvest, we sequenced their bacterial and fungal rhizosphere microbiomes using 16S and ITS sequencing, respectively. Using a custom-built root imaging platform, we quantified the root architectural traits specific root length (SRL), root tissue density (RTD), diameter, the ratio of fine to coarse roots (fine:coarse), and measured root nitrogen and carbon content. We then applied Bayesian phylogenetic mixed-effects models to quantify variance explained by host phylogeny versus species identity on microbiome composition. Lastly, we used machine learning to quantify the explanatory power of root traits and species identity for rhizosphere community composition.

## Materials and methods

### Selection and growing plants

In this study, we focused on legumes (Fabaceae) because they contain many globally important crop species, exhibit high diversity in root architecture, and span a long evolutionary history within a single plant family (Table S1) [48–50]. Legume plant species in this study include: *Arachis hypogea, Cicer arietinum, Cicer reticulatum, Glycine max, Glycine soja, Lens culinaris, Lupinus albus, Medicago sativa, Medicago truncatula, Phaseolus coccineus, Phaseolus vulgaris, Pisum sativum, Pisum sativum elatius, Vicia sativa, Vigna unguiculata*. Within the legumes, the design incorporated three crop-wild relative pairs: *Pisum sativum* and *Pisum sativum elatius*, *Cicer arietinum* and *Cicer reticulatum*, and *Glycine max* and *Glycine soja*. We also included seven non-legume outgroup species for comparison (*Arabidopsis thaliana, Brachypodium distachyon, Brassica oleracea, Cucumis sativus, Cucurbita pepo, Solanum lycopersicum, Zea mays*). Plants were then germinated in standardized soil (*SM text: Soil collection/germinating plants*).

Low and high phosphorus treatments (Low-P, High-P) were administered using Hoagland solutions, with Low-P receiving ~21-fold less plant-available phosphorus than the High-P condition over the duration of the experiment. All plants were supplied with 100 ml modified Hoagland immediately after transplantation to the pot, and then weekly until harvest after 21 days (*SM text: Hoaglands*). Each plant species had 24 replicates, with 12 plants per Low-P and High-P treatment and were grown in a greenhouse environment in standardized soil (*SM text: Greenhouse*). Upon harvesting rhizosphere soil (*SM text: root processing*) was collected for molecular analysis. Root systems were then imaged using a custom-built imaging platform designed to ensure consistent imaging conditions, following the design of Seethepalli 2020 [53], (*SM text: root imaging*). Root images were then processed using Rhizovision software to calculate architectural traits including diameter, RTD, SRL, and the ratio of fine to coarse roots (“SM: root traits”).

### Quantifying microbial community composition and drivers of variation

Rhizosphere DNA was extracted using the MagAttract PowerSoil Pro DNA extraction kit (Qiagen, USA), on a KingFisher robot (Thermo-Scientific, USA), following the manufacturer’s protocol. We then sequenced DNA using the NovaSeq 6000 platform (PE 2 × 250), targeting the V3–V4 region of the bacterial 16S rRNA gene and the ITS2 region for fungi, and processed using the Lotus2 pipeline (“SM text: DNA sequencing”, Fig. S6–8). These regions are widely used for general characterization of microbiomes, but are known to have poor taxonomic resolution for rhizobia and arbuscular mycorrhizal fungi [54, 55].

To test whether plant identity and phosphorus treatment influence overall microbial community composition, we summarized bacterial and fungal communities by calculating Bray–Curtis dissimilarity on ASV tables. PERMANOVA and tests of beta dispersion (*n* = 999 permutations for both) were performed to evaluate the effects of plant identity and phosphorus treatment on both community composition and variation in dispersion with the package “vegan” [56]. We then used a series of mixed-effects models to test the effects of host species and phylogeny on microbiome composition. These models use intraclass correlation coefficients (ICCs) to quantify the proportion of variance explained by species identity or phylogeny, allowing direct comparison of their relative contributions (“SM text: Bayesian models”).

### Machine learning modeling of rhizosphere community composition with root traits

To first describe and visualize how variation in root traits relates to differences in rhizosphere community composition across plant species we used an ordinations. Principal Coordinates Analysis (PCoA) of Bray–Curtis dissimilarity of root traits (diameter, RTD, SRL, fine:coarse roots, carbon %, and nitrogen%) was conducted. Trait values were then projected onto the ordination to identify which root traits contribute to variation between plant species using the “envfit” function in “vegan” [56]*.* PERMANOVA tested effects of species, traits, and phosphorus on community composition.

To further test relationships between root traits and patterns in microbial community, we applied a machine learning framework. We fitted models using the Random Forest algorithm on the first two PcoA axes for both bacterial and fungal communities as functions of root traits and species identity, using leave one out cross-validation (“SM: Machine learning”). We then used the SHAP algorithm to quantify how root trait values impact model estimations [57].

### The impact of domestication on the rhizosphere and root traits

Domestication effects were assessed using three crop species and their wild relatives (*Pisum, Cicer, and Glycine*). To do so, the dataset was subset to include only the paired wild and domesticated species, and microbial community composition was analyzed using Bray–Curtis dissimilarities. Community composition was visualized with PCoA, tested using a PERMANOVA, stratified by plant genus. Beta dispersion permutational tests were used to assess within-group variance.

Root trait differences between wild and domesticated plants were tested using linear mixed-effects models fit with the “lme4” package [58]. For each trait, models included domestication status, genus (species pair), phosphorus treatment, and their interaction as fixed effects. Greenhouse block was included as a random intercept to account for experimental design. Estimated marginal means and pairwise contrasts were calculated using the “eemeans” package to compare wild and domesticated plants within each genus, with Benjamini-Hochberg correction [59].

## Results

### Variance explained by host phylogeny and species identity

We found that for both bacterial and fungal community composition, rhizosphere communities varied significantly by plant species (Fig. 1B and C, PERMANOVA; bacteria: R^2^ = 0.44, *P* < .001; fungi: R^2^ = 0.40, *P* < .001). In contrast, phosphorus treatment had a weaker effect on community composition (PERMANOVA; bacteria: R^2^ = 0.01, *P* < .001; fungi: *P* = .18). Multivariate tests of dispersion indicated significant differences in dispersion among plant species and phosphorus treatment for both bacteria and fungi (*P* < .001).

Using Bayesian mixed-effects models, we then estimated phylogenetic signal and variance explained by species identity independent of phylogeny using ICCs. ICCs represent the proportion of variance in community composition explained by a random effect in a mixed-effects model. Models were fitted first on two axes of a PCoA analysis based on Bray–Curtis dissimilarity for both PC1 (bacteria = 19.9%, fungi = 24.4%) and PC2 (bacteria = 8.9%, fungi = 14.3%). Compared to bacterial models that included only phosphorus treatment or greenhouse location, which performed poorly (mean WAIC = −1203.0 ± 180.0; R^2^ = 0.01), models incorporating species (mean WAIC −1896.0 ± 257.0; R^2^ = 0.75), phylogeny (mean WAIC −1892.0 ± 258.0; R^2^ = 0.75), or both (mean WAIC −1895.0 ± 257.0; R^2^ = 0.75) showed substantially improved fit and explained far more variance across both PCoA axes (Table S2). Similarly, for fungal community composition along both PCoA axes tested, models including only phosphorus treatment or greenhouse block had the weakest performance (mean WAIC = −315.0 ± 137.0; R^2^ = 0.01). Fungal models that included species (mean WAIC −668.0 ± 24.5; R^2^ = 0.51), phylogeny (mean WAIC 665.0 ± 26.2; R^2^ = 0.51), or both species and phylogeny (mean WAIC −668.0 ± 24.5; R^2^ = 0.51) provided substantially better fits and explained more variance of community composition (Table S3).

For bacterial community composition, we found species effects had higher ICC compared to the phylogeny random effect (Fig. 1D). Species identity effects were strongest on PC1 (Mean ICC = 0.71 ± SD 0.12), while phylogenetic effects on community composition were weaker (ICC = 0.13 ± 0.12). A similar pattern was observed for PC2, where species identity explained more variance (ICC = 0.79 ± 0.10) compared to phylogeny (ICC = 0.15 ± 0.14). Similar to bacteria, species identity explained more variance in the composition of the fungal community than host phylogeny (Fig. 1E, PC1: Species = 0.61 ± 0.15, Phylogeny = 0.06 ± 0.05; PC2: Species = 0.40 ± 0.12, Phylogeny = 0.02 ± 0.01).

### Impact of root traits on microbiome community composition

**Figure 2:**
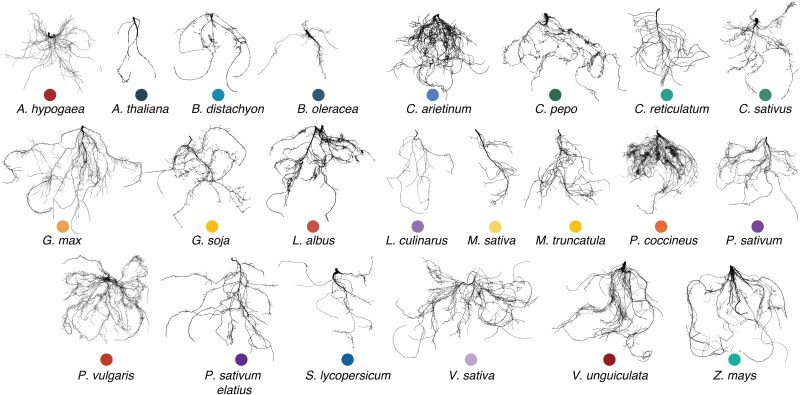
Root system imaging examples from this study. Roots were harvested immediately after rhizosphere sampling and imaged using a custom-built imaging platform. Roots were carefully spread prior to imagine to minimize overlap where possible, though some overlap was unavoidable—a common limitation in root phenotyping which may influence root trait calculations. Images were processed with RhizoVision to calculate the architectural root traits analyzed in this study (see Methods). The color of each dot matches plant species identity in other graphs for direct comparison. Images with scale bars are available on our GitHub repository, as differences in root size across species make it impractical to present them consistently here.

We found that root traits significantly varied by plant species (Fig. 2 and Fig. S1B, R^2^ = 0.68, *P* < .001), with a weak effect of phosphorus treatment (R^2^ = < 0.01, *P* = .002; PERMANOVA, stratified by greenhouse location). Tests of homogeneity of variance indicated significant differences in trait value variance across both phosphorus treatment and plant species (all *P* < .001), thus these results should be interpreted with caution [60].

Next, using the Bayesian modeling approach applied to rhizosphere communities we compared the variance explained between species effects and phylogenetic signal for root traits. Across all traits, models including species, phylogeny, or both random effects outperformed those with only phosphorus treatment and greenhouse block, by comparing WAIC scores (Fig. S2, Table S4). These models explained substantially more variance (across traits R^2^ = 0.15–0.74, Fig. S3).

Root traits varied in the strength of phylogenetic signal and species effects, but in all cases species effects explained more variance than phylogenetic effects. The variance explained by the random effects (ICC ± SD) was highest for nitrogen content (Species = 0.55 ± 0.26, Phylogeny = 0.05 ± 0.03), followed by the ratio of fine:coarse roots (Species = 0.54 ± 0.12, Phylogeny = 0.90 ± 0.1), average root diameter (Species = 0.54 ± 0.18, Phylogeny = 0.04 ± 0.03), SRL (Species = 0.41 ± 0.16, Phylogeny = 0.02 ± 0.01), root carbon % (C: Species = 0.35 ± 0.21, Phylogeny = 0.02 ± 0.01), and RTD (Species = 0.14 ± 0.09, Phylogeny = 0.01). These results indicate that root trait variation was primarily driven by species-specific differences rather than shared evolutionary history.

Next, to correlate rhizosphere community composition with plant root traits, we used Random Forest models, while accounting for differences in phosphorus treatment and greenhouse location. Root traits alone explained 57.1% (for PC1) and 44.9% (for PC2) of the variance in bacterial community composition and 46.0% (for PC1) and 11.6% (for PC2) of the variance in fungal community composition. To assess whether plant species identity explained additional variance beyond root traits, Random Forest models were refitted with plant identity included alongside root traits. Plant identity explained an additional 0.13 of variance (total R^2^=0.71) and 0.35 of variance (total R^2^=0.80) in bacterial PC1 and PC2, respectively, and 0.10 of variance (total R^2^ = 0.56) and 0.33 of variance (total R^2^ = 0.45) in fungal PC1 and PC2, respectively. Using leave-one-out cross-validation, these models showed strong agreement between observed and predicted PC scores for both bacterial and fungal communities (Fig. S4).

**Figure 3:**
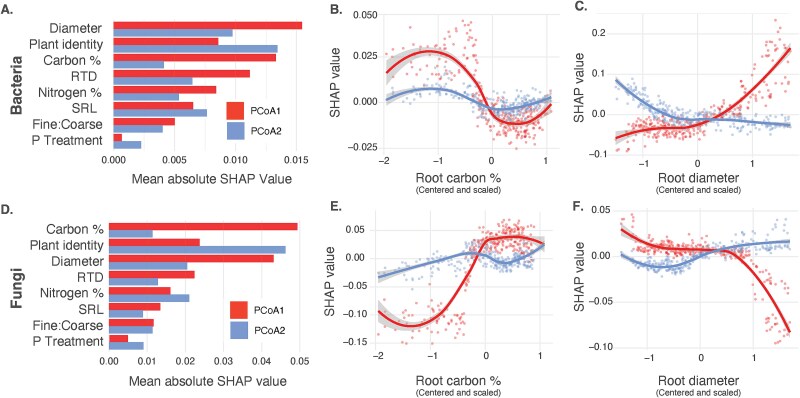
Results of Random Forest models correlating plant identity and root traits with rhizosphere composition for both bacteria and fungi. Community composition was summarized using principal coordinates analysis on Bray–Curtis dissimilarity. Random Forest models were fitted to predict variation in the first two PC axes of bacterial and fungal communities using either root traits only or a combination of root traits and plant species identity. This approach allowed for the quantification of variance in microbiome composition explained by root traits and the assessment of additional variance explained by species identity that was not captured by root traits alone. For bacterial communities, PC1 and PC2 explained 19.9% and 8.9% of total variation, respectively, while for fungal communities, PC1 and PC2 explained 23.7% and 14.3%, respectively. In the Random Forest models for bacteria, the root traits-only models achieved R^2^ = 0.57 (PC1, RMSE = 0.05) and R^2^ = 0.45 (PC2, RMSE = 0.05), while the combined root traits and plant identity models achieved R^2^ = 0.70 (PCoA1, RMSE = 0.05) and R^2^ = 0.80 (PC2, RMSE = 0.03). For fungi, the root traits-only models achieved R^2^ = 0.46 (PC1, RMSE = 0.15) and R^2^ = 0.12 (PC2, RMSE = 0.16), while the combined root traits and plant identity models achieved R^2^ = 0.56 (PC1, RMSE = 0.15) and R^2^ = 0.44 (PC2, RMSE = 0.14). Variable importance was quantified using the Shapley algorithm to produce SHAP values, which show the relative contribution of each predictor to the model (A, D), and SHAP values were also used to visualize the marginal effects of key predictors, including root carbon (%) (B, E) and root diameter (C, F).

We conducted a SHAP analysis to estimate the marginal effect of individual predictors to variation in rhizosphere composition (Fig. 3A and B). Positive SHAP values indicate that higher values of a predictor are associated with increases in the response variable (e.g. PCoA axis score), while negative SHAP values indicate that higher predictor values are associated with decreases in the response. The absolute SHAP value reflects the overall importance of a predictor, with larger absolute values indicating stronger contributions to model predictions, regardless of direction. Root diameter (absolute SHAP value: bacteria PC1 = 0.015, PC2 = 0.010, fungi PC1 = 0.043, PC2 = 0.020) and root carbon % (absolute SHAP value: bacteria PC1 = 0.013, PC2 = 0.004, fungi PC1 = 0.049, PC2 = 0.011) were identified as two of the most important root trait predictors of PC scores. Beyond root traits, plant identity was also identified as an important predictor of microbiome composition (absolute SHAP value: bacteria PC1 = 0.009, PC2 = 0.013, fungi PC1 = 0.024, PC2 = 0.046). The relationship between root traits and PC axes for both bacteria and fungi were nonlinear (Fig. 3B–F).

### Impact of domestication on rhizosphere communities and root traits

**Figure 4:**
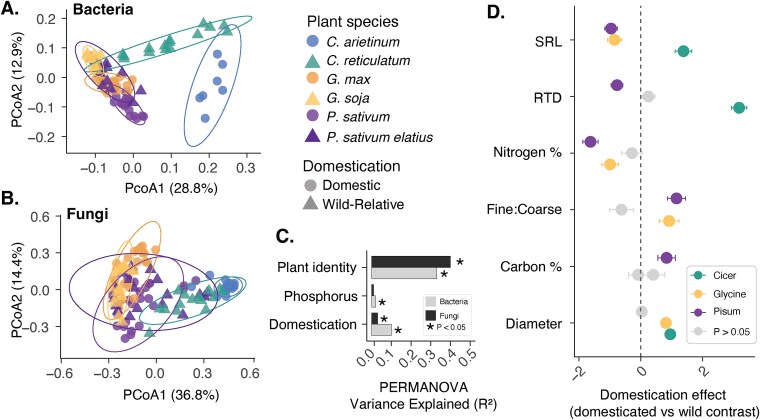
Impact of domestication on rhizosphere communities and root traits. Three pairs of crops and wild relatives (crop = *C. arietinum*, wild relative = *C. reticulatum*), (crop = *G. max*, wild relative = *G. soja*), (crop = *P. sativum*, wild relative = *P. sativum elatius*) were assessed. (A) PCoA of bacterial communities based on Bray–Curtis distances, with circles representing domesticated plants and triangles representing wild relatives. (B) Same as (A) but for fungal communities. (C) PERMANOVA analysis showing R^2^ values for explanatory variables (plant identity, phosphorus treatment, domestication status); dark bars represent fungi, light bars represent bacteria. (D) Coefficients from linear mixed-effects models comparing root traits between wild and domesticated plants; points indicate mean estimates with error bars representing standard error, and grey indicates non-significant comparisons (*P* > .05).

Crop domestication was associated with measurable shifts in both bacterial (Fig. 4A–C, PERMANOVA, R^2^=0.10, *P* = .001) and fungal (R^2^=0.03, *P* = .001) community composition, although these effects were relatively small. In this subset of species, we found a larger proportion of variation was explained by plant species identity for bacterial (R^2^=0.33) and fungal (R^2^=0.40) community differences (*P* = .001 for both). Phosphorus treatment had a minor but significant effect on bacterial composition (0.02, *P* = .002), while its effect on fungi was weaker and not significant (0.01, *P* = .081). Tests of homogeneity of variance indicated significant differences in microbial community dispersion across plant species (*P* = .001 for both bacteria and fungi), but not between domestication statuses (*P* = .8 for bacteria, *P* = .56 for fungi) or phosphorus treatments (*P* = .14 for bacteria, *P* = .43 for fungi). PC scores showed no convergence toward a single domesticated community, with clustering instead driven by plant genus (Fig. 4A and B).

To test for domestication effects on root traits, we used linear mixed-effects models and computed relevant effect sizes using estimated marginal mean contrasts. For five out of the six root traits (all except root carbon concertation) we found evidence for genus-specific effects of domestication status on root traits (Fig. 4D mixed effects ANOVA, Genus x Domestication status interaction *P* < .05, Table S5, Fig. S5). This significant interaction arose either due to different genera showing different directions in the domestic-wild contrast, or a combination of non-effects and positive or negative changes. For RTD, domesticated *Cicer* showed a strong increase (+3.18 SD, *P* < .05), while domesticated *Pisum* had lower RTD than its wild relative (−0.77 SD, *P* < .05), and *Glycine* showed no significant difference. Similarly, SRL was higher in domesticated *Cicer* (+1.38 SD, *P* < .05), but lower in domesticated *Glycine* (−0.85 SD, *P* < .05) and *Pisum* (−0.96 SD higher, *P* < .05).

Domestication was associated with increased average root diameter in *Cicer* (+0.96 SD, *P* < .05) and *Glycine* (+0.82 SD, *P* < .05), whereas no difference was detected in *Pisum*. Compared to wild relatives, domesticated species showed higher fine:coarse root ratios in *Pisum* (+1.16 SD, *P* < .05) and *Glycine* (+0.92 SD, *P* < .05), with no significant difference in *Cicer*. Nutrient-related traits showed the opposite trend, with domesticated plants exhibiting lower root nitrogen concentrations in *Glycine* (−0.99 SD, *P* < .05) and *Pisum* (−1.63 SD, *P* < .05), and no significant difference in *Cicer*. Only root carbon concentration showed a significant effect of domestication status without a corresponding significant Genus x Domestication interaction. The difference between wild and domesticated plants, averaged over the three genera, was +0.46 (marginal effect, domestic estimate = 0.218, wild estimate = −0.239). Root carbon concentration was significantly higher in domesticated *Pisum* (+0.84 SD, *P* < .05), while no significant differences were observed in *Cicer* or *Glycine*.

## Discussion

By analyzing the rhizosphere microbiomes of >500 individual plants, we found that host phylogeny explained little of the variance in rhizosphere community composition for both bacteria and fungi, compared to species identity (Fig. 1). Leveraging root trait data and machine-learning we were able to predict species-level variation in rhizosphere communities and identified root diameter and carbon % as being the most important predictors (Figs 2 and 3). Wild and domesticated plants showed that domestication effects were weak and varied by plant genus (Fig. 4). Together these results suggest that, within a single plant family, host relatedness is a poor predictor compared to root traits for microbiome assembly.

### Weak phylogenetic signal in rhizosphere community composition

We found evidence that host phylogeny is largely uncorrelated to rhizosphere community composition (Fig. 1D and E). While model comparison failed to identify the single best model comparing phylogeny and species effects, species effects explained over half of the variation in both bacterial and fungal community composition. These results align with other research that also found limited phylogenetic signal in rhizosphere communities across angiosperms [26]. In contrast, research across 25 plant families has identified core microbial groups conserved by host plants [21]. Core microbiomes vary in definition but are commonly defined as the microbial taxa shared across two or more samples from a particular host or environment [61]. The concept of a rhizosphere core microbiome remains debated, as even putative members may not be tightly linked to host evolution [61, 62]. For example, experimental work found that rapid evolution of bacterial mutualisms can occur in the plant rhizosphere—*Pseudomonas protegens* evolved beneficial traits for *Arabidopsis thaliana* within just six plant generations [63]. Such rapid evolution could work against the development of long-term co-evolutionary patterns with hosts, as fast microbial adaptation may continually shift community composition. Additionally, horizontal gene transfer among rhizosphere bacteria may further complicate the detection of clear phylogenetic signals, as functional genes critical for plant interactions may rapidly transfer across taxa [64].

We also found that correlations between microbiome composition with plant hosts were stronger for bacterial communities than for fungi. Similar patterns have been observed for other plant hosts, where correlation of host phylogeny or identity with fungi are relatively weak or even absent [18, 31]. In contrast to rhizosphere communities, endosphere microbiomes, often but not always, show tight correlations to plant evolutionary history, including fungi [26, 65–68]. Our findings suggest the assembly of rhizosphere communities varies not only across plant species within a single plant family, but also between different kingdoms of microbes. However these differences may be due in part to lower sampling depth and incomplete saturation of species richness across some host species (Fig. S7).

The comparative lack of phylogenetic signal in the rhizosphere may be explained by the fact that endosphere microbes are often compartmentalized within host tissues (e.g. rhizobia). Such compartmentalization allows plants to exert greater control over microbial partners, promoting co-evolution through mechanisms like isolation of symbionts and exclusion of non-beneficial strains [69]. In contrast, the rhizosphere is an open environment dominated by free-living microbes, where interactions with hosts may be more transient. Lack of monitoring and control by host plants can limit co-evolution because, without selective pressures imposed by the host, microbes are not consistently rewarded for benefits conferred to hosts or punished for being ineffective [69, 70]. Further, rhizosphere microbes are also under strong selection to maximize their own fitness, potentially at the plant’s expense [41, 71]. For example, microbes can stimulate increased root exudation of amino acids, enhancing rhizosphere microbes access to carbon resources while imposing metabolic costs on the host plant [72, 73]. Such reduced host control in the rhizosphere, and the rapid evolution of microbes themselves, may in fact limit the extent of co-evolution between host plants and rhizosphere communities.

### Root traits correlate with rhizosphere

Using machine learning, we were able to model rhizosphere community composition with high accuracy for both bacteria and fungi, relying primarily on species-specific variation in root traits, particularly root diameter and carbon content (Fig. 3). These correlations held despite being measured across contrasting nutrient treatments, which are known to influence both root architecture and microbiome composition [39–41]. Such correlations suggest that the relationship between root traits and microbiome assembly may be predictable, in soil conditions relevant for farmers (e.g. change in nutrient conditions through fertilizer application). Our results, along with other research, suggests that root traits may be helpful in predictive frameworks for engineering of crop microbiomes [31, 32].

We also found root trait variation is largely independent of host relatedness history. This may be explained by our study’ focus on a single plant family, with four outgroup plant families represented. The inclusion of these outgroups likely facilitated comparisons of microbiome and plant traits since our study focused on legume crops. In contrast, other studies comparing more distantly related plant lineages across multiple plant families have identified strong phylogenetic signals in root trait organization across plant lineages [42]. Together, these findings suggest that the predictive power of host phylogeny for microbial composition is scale-dependent, with stronger effects observed between plant families than within them.

The most influential root traits identified in modeling rhizosphere community composition, root carbon % and diameter, align closely with the plant economic spectrum [74, 75]. The plant economic spectrum is a framework to explain traits related to resource acquisition and use vary among different plant species. Plants allocate fixed carbon to different resource acquisition strategies, balancing investment in “cheap” fast-growing, thin roots with shorter lifespans, versus more “expensive”, thicker roots built for longevity and often greater reliance on mycorrhizal fungi [74–76]. Simultaneously, a substantial portion of carbon, estimated at 5%–21%, is directed toward root exudates, which shape the rhizosphere microbial community through signaling [33, 76–78]. Because the plant’s carbon budget is partitioned across competing demands, correlations with root traits may reflect underlying trade-offs in carbon allocation along the plant economic spectrum [75]. Such tradeoffs in carbon allocation may therefore complicate determining if root traits themselves drive patterns of rhizosphere community composition or are merely proxies, e.g. for root exudation profiles—recent research on grasses has correlated root exudation rates and composition with root architecture and found that thicker roots had faster exudation rates [30].

Moreover, rhizosphere microbes themselves influence plant root architecture; for instance, *Variovorax* species can suppress root growth stimulated by other microbes, and other bacterial taxa can manipulate hormone signaling to alter lateral root development [40, 41]. However, our findings provide further evidence that the plant economic spectrum extends beyond mycorrhizal symbioses and likely influences broader rhizosphere microbial communities, including bacteria and free-living fungi [75, 79].

### Domestication and rhizosphere communities

Crop domestication was associated with measurable but modest shifts in rhizosphere microbial communities (Fig. 4C). We found no evidence of convergence toward a common “domesticated microbiome”. Instead, shifts in microbial diversity were highly genus-specific, aligning with findings from recent meta-analyses [80]. Further we found that domestication explained only ~10% of bacterial community variation and even less (~3%) for fungi. Modest effects of domestication on community composition, comparing crop bacterial microbiomes with their wild relatives have also been observed for other crops including maize, wheat, and common bean, however the effect size varied between different genera [80].

Such shifts in rhizosphere composition likely arise from multiple processes acting simultaneously. First, domestication-induced changes in root architecture can alter a plant’s capacity for independent nutrient uptake [81]. Given that our analyses suggest root traits play a role in structuring rhizosphere microbial communities, alterations in root architecture likely contribute to observed divergence in rhizosphere microbial diversity between domesticated plants and their wild relatives. Further, because domestication involves artificial selection targeting plant traits beneficial to humans rather than aligning directly with plant fitness, domesticated hosts may exert reduced control over their rhizosphere communities [13, 82].

Our results add to a growing body of evidence showing that plant domestication influences the composition of rhizosphere microbial communities [83–88]. In this study, we examined domestication effects using only three crop-wild relative pairs, each represented by a single genotype, and we therefore recognize that genotypic variation also plays an important role in shaping rhizosphere microbial communities [9, 89]. In addition how much domestication has impacted rhizosphere microbial communities are likely biased by the soils and climate in which experiments are conducted. Soil origin is a critical factor explaining variation in the rhizosphere microbial communities across plant species [9, 17]. Major drivers of this soil-origin effect include the composition of plant communities themselves, as well as climate and soil chemistry [90–92]. Comparisons of crops and their wild relatives across multiple soil contexts are likely necessary—including soils in centers of origin, regions of intensive cultivation outside centers of origin, and areas where these plants are rarely grown [93]. Without accounting for the influence of soil origin and plant genotype, attempts to design microbiome-informed breeding strategies may overlook key ecological contexts that shape plant-microbe interactions.

### Implications and future directions

Our findings suggest that within a single plant family, host phylogeny has a limited role in shaping bacterial and fungal rhizosphere communities. Instead, species identity and root traits explained more variation in rhizosphere community composition. The mechanisms underlying these relationships remain unclear, such as whether root architectural traits directly shape rhizosphere assembly or whether they act as proxies for underlying differences in root exudation. It is also unknown which specific microbial taxa are most strongly associated with individual root traits. Research examining how plants allocate carbon between structural investment in roots and the production of root exudates is needed to address these uncertainties.

Future studies would also benefit from expanding the scope of sampled microbial groups to include rhizobia and arbuscular mycorrhizal fungi. For example, while rhizobia nodules were found across all but five legume hosts (Figs S9 and S10), the lack of strain-level resolution limits our ability to assess functional variation in symbiosis. Such potential artifacts may contribute to differences in root nitrogen content and, in turn, influence broader patterns of rhizosphere community composition [41, 94, 95]. Furthermore, these symbiotic associations with plant hosts develop over time and may be strongly influenced by plant developmental stage and the timing of symbiosis formation [96]. Study designs that focus on specific symbiotic relations, and compare them with field observations would therefore be needed for truly robust interpretations. Further, while soil conditions were standardized in this experiment, it remains possible that variation in seed endophytes contributed to differences in rhizosphere community composition. This highlights the need to explicitly examine the relative contributions of seed borne versus soil derived microbes to rhizosphere recruitment and assembly [97, 98].

Lastly, we found that domestication associated shifts in rhizosphere communities were statistically significant but relatively weak. This suggests that root traits contribute to domestication effects but do not fully explain differences in microbial community composition between crops and their wild relatives. Future research should test whether these effects depend on contrasts between agricultural soils and native soils at crop centers of origin, as well as variation across multiple genotypes and seed sources.

## Supplementary Material

SM_Stewart_etal_ISMECOMMS_Accepted_Submit_ycag087

TableS2_ycag087

TableS3_ycag087

TableS4_ycag087

## Data Availability

All data and code is available on Github. Sequences generated in this study have been deposited in the NCBI SRA (SRR34418801 and SRR34423936).
