# Supplementary material for: Root traits correlate with crop rhizosphere microbiome diversity independent of legume relatedness
Source: ISME Commun. 2026 Apr 14;6(1):ycag087. doi: 10.1093/ismeco/ycag087 (PMC13155103; doi:10.1093/ismeco/ycag087)
Supplement: SM_Stewart_etal_ISMECOMMS_Accepted_Submit_ycag087 [file sm_stewart_etal_ismecomms_accepted_submit_ycag087.docx]

**Supplemental Text:**

*Soil collection and germinating plants:*

We rinsed seeds with deionized water and 70% ethanol before germination (Table S1). Since plants germinated at different speeds, we planted them in three cohorts to ensure a uniform growth period before harvesting after 21 days. We acknowledge that plants may have different growth rates and aimed to minimize confounding effects of this using cohorts. The cohorts were - Fast germinators: *Cicer arietinum, Lens culinaris, Lupinus albus, Medicago sativa, Medicago truncatula, Phaseolus vulgaris, Pisum sativum, Vigna unguiculata*. Intermediate germinators: *Arabidopsis thaliana, Brassica oleracea, Cicer reticulatum, Cucumis sativus, Phaseolus coccineus, Solanum lycopersicum, Vicia sativa, Zea mays.* Slow germinators: *Arachis hypogea, Brachypodium distachyon, Cucurbita pepo, Glycine max, Glycine soja, Pisum sativum elatius.* Seedlings were transplanted into 1.5 liter pots lined with fine nylon mesh and filled with 1 L of moist soil (1.12 kg dry soil). We acknowledge that this pot size may constrain root system expansion for larger or deeper rooting species, potentially masking differences in root architecture. We used collected soil from an organically managed agricultural field in Nergena, Bennekom (coordinates: 51.996250, 5.659375). In 2014, collaborators in the [MiCRop research consortia](https://www.microp.org/) excavated the top 80 cm of soil and stored it outdoors without management, allowing a natural soil community to persist through the growth of wild plants. Before use in experiments, we air-dried batches of soil, sieved through a 5 mm mesh to remove larger particles and roots, and then stored in the dark. As a control we use soils with no plant host.

*Host phylogeny:*

A phylogenetic tree of the host plant species in this study was generated using package *V.PhyloMaker2,* which provides a time-calibrated phylogeny of vascular plant species [1]. From this backbone, we pruned a phylogeny including the 22 species used in our experiment, spanning approximately 135 million years of evolutionary history. All species were present in the backbone except for *Arachis hypogaea*, *Glycine soja*, and *Pisum sativum elatius*, which were substituted with the closely related congeners *Arachis major*, *Glycine latifolia*, and *Pisum abyssinicum*, respectively, based on their placement within the same genus.

*Hoaglands:*

A modified Hoaglands was used that contained 5.6 mM NH4NO3, 0.8mM MgSO4 x 7 H2O, 0.8mM KSO4, 1.6 mM CaCl2 x 2 H2O, 40 µM FeSO4 x 7 H2O, 40 µM EDTA.Na2 x 2 H2O, 11.45 µM H3BO3, 2.25 µM MnCl2 x 4 H2O, 0.15 µM CuSO4 x 5 H2O, 0.7 µM ZnCl2, 0.05 µM Na2MoO4 x 2 H2O. For the High P treatment 0.8 mM K2HPO4 x 3 H2O was added. For Low P treatment 1.6 mM KCl was added instead, and pH was adjusted to 7.0. The plant-available P in our soil was 0.5 mg kg^-1^ dry soil. For each pot, approximately 1.12 kg dry soil was used to a total of 0.56 mg plant-available P in each pot in the soil. Total plant-available P in the Low P treatment was 0.56 mg per pot originating from soil alone. Total plant-available P in the High P treatment was ~10.0 mg P per pot from the Hoagland additions from four total applications). Plant pots were checked every 3 days and water was added as needed (i.e. if topsoil was dry).

*Greenhouse:*

We grew plants across 11 greenhouse blocks, with each block randomized to include representatives of every species and both phosphorus treatments to control for spatial variation within the greenhouse environment (1120 umol/second/m^2^, 16h day 8h night). Control plots were also included without plants (Figure S8).

After growing, we harvested plants in the order of planting cohorts, and within each cohort, we harvested by species and then by treatment. Shoots were cut just above the soil surface, placed in paper bags, and dried at 70°C for one week. To extract root systems, pots were carefully inverted, and the soil containing the roots was collected in a tray sprayed with 70% ethanol. Loosely adhering soil was removed manually. To prevent cross-contamination, gloved hands, trays, and utensils were sprayed with 70% ethanol between plants, and gloves were changed between treatments and species. Roots were transferred to 50 mL tubes with 25 mL of deionized water, shaken by hand for one minute to collect rhizosphere samples. The rhizosphere suspension was immediately stored on ice and frozen at -20°C until further processing for DNA extraction. Control pots were sampled by taking a soil sample from the center of each pot.

*Root processing:*

Fresh root mass was recorded for each sample and a subsample of known wet mass was then dried at 70°C for at least 48 hours to determine its dry mass. Using this subsample root wet-to-dry mass ratio was calculated for each plant. This ratio was applied to estimate total dry biomass for the entire root system. The dried subsample was subsequently ground for elemental analysis. Carbon (C) and nitrogen (N) concentrations were measured via dry combustion using an elemental analyzer (Flash EA 1112, Thermo Scientific, Rodana, Italy). For phosphorus (P) content, a 50 mg portion of the ground material was digested in 1 mL of a 1:4 mixture of 37% (v/v) HCl and 65% (v/v) HNO₃ in a closed Teflon cylinder for 6 hours at 140°C. After digestion, samples were diluted with 4 mL demineralized water, and total P was quantified spectrophotometrically using the ammonium molybdate method (Murphy & Riley, 1962).

*DNA sequencing:*

DNA was extracted using the MagAttract PowerSoil for Kingfisher, following manufacturer instructions and quantified using a NanoDrop spectrophotometer. All samples passed quality control criteria. DNA was then sequenced at BGI Genomics (Hong Kong) on a NovaSeq 6000 platform (PE 2 × 250), targeting the V3–V4 region of the 16S rRNA gene and the ITS2 region for fungi. Sequencing depth was approximately 100,000 reads per sample. For computational efficiency, data were subsampled to 100,000 reads per sample using *vsearch* function “fastx_subsample” [2]. Rarefaction curves were saturated after subsampling, indicating that community diversity was preserved at a smaller sample size (Figure S6-7). All downstream analyses were conducted on these subsampled datasets. Raw sequence data for both 16S and ITS were processed using the Lotus 2 pipeline, following the recommended operating protocols[3–5]. Sequencing quality was first assessed, followed by denoising with the DADA2 algorithm to generate amplicon sequence variants (ASVs) [6].

For 16S, a total of 53,700,000 raw reads were obtained, which were filtered to 45,269,006 reads, yielding 31,964 ASVs. For ITS, 48,131,929 raw reads were obtained, which were filtered to 28,295,720 reads using ITSx, yielding 5,563 ASVs [7]. Taxonomy for both datasets was assigned using the Lambda classifier, with SILVA (SLV_138.1_SSU.fasta) for 16S and the UNITE database for ITS (v9_25.07.2023)[8]. Results from Lotus2 were then exported as phyloseq objects for use in R (4.4.2) [9–11]. Potential contaminants were removed using the prevalence method implemented in the decontam package using water samples from both laboratory and greenhouse environments as negative controls [12].

*Root imaging:*

We constructed a root imaging platform following the design of the Rhizovision Crown platform. A high-resolution digital camera (Canon EOS 2000D, 0.08 mm/pixel.) was fixed to a stable frame above a tray used for root imaging. Roots were floated in water within the tray and carefully disentangled to minimize overlap as much as possible. A uniform LED panel (30 × 30 cm²) illuminated roots to minimize shadows and ensure consistent contrast, enhancing fine-root visibility. A custom graphical user interface developed in MATLAB 2022b controlled the camera and lighting system, saved images, and allowed for image quality preview. An Arduino UNO triggered the LED lighting. To prevent sample mislabeling, each plant pot was assigned a unique ID, scanned using a barcode scanner connected directly to the imaging computer.

*Root traits:*

Root traits were selected based on their relevance to plant nutrient uptake strategies and their roles within the plant root economics spectrum, which describes trade-offs between resource acquisition and conservation strategies in roots [13–15]. We measured key traits of the root economics spectrum: specific root length (SRL), which measures root length per unit dry mass (m g⁻¹), root tissue density (RTD) through dry mass per unit volume (g cm⁻³), root nitrogen percentage, root carbon percentage. We also measured root diameter (cm), root volume (cm^3^, and root surface area (cm^2^) to capture overall root system size. Root lengths were then separated into fine (< 2mm) and coarse (>2mm) roots and the ratio of fine:coarse roots was calculated. Root nodulation was visually assessed for each plant and recorded as a binary presence-absence variable; however, nodulation was inconsistent across replicates and was therefore excluded from subsequent analyses (Figure S9). Subsequent analyses were conducted in R (4.4.2).

Root traits are often highly correlated with one another, as many traits are linear transformations of shared structural properties (e.g., root volume scales with overall root size) [15]. This can introduce multicollinearity, complicating efforts to isolate the effects of individual traits on microbiome composition. Multicollinearity among traits was assessed using variance inflation factors (VIF) with the *car* package [16]. We found high collinearity among surface area, total length, volume, and SRL. We retained SRL as a representative trait due to its widespread use in root trait studies. The final root traits we included were SRL, root carbon concentration (%), root tissue density (RTD), root nitrogen concentration (%), and the fine-to-coarse root ratio. We scaled and centered all traits prior to analysis to ensure comparability across trait types and species. We also removed outliers above the 95^th^ percentile of values per trait for all analyses.

*Machine learning:*

Advances in machine learning have enabled nuanced analysis of how host traits influence microbial community composition [17–20]. Such approaches allow for the identification of nonlinear relationships, quantification of variable importance, and the estimation of the marginal effects of traits from machine-learning models.

Models were first fitted with root traits and soil phosphorus treatment only, and then refitted with root traits, phosphorus treatment, plus one-hot encoding of plant species identity. Optimal model parameters were selected based on minimizing root mean square error (RMSE) and maximizing R² on held-out folds using leave-one-out (LOO) cross validation. Under LOO cross validation, each model iteration is trained on all samples except one, and model performance is evaluated by predicting the held-out observation. This process is repeated across all samples, ensuring that every observation serves as an independent test case and that no sample is ever evaluated using a model trained on itself. Model performance was compared based on R² and RMSE to evaluate the additional explanatory power of species identity beyond variation captured by root traits. Models were implemented in the *caret* package with *ranger [21, 22]*. Residuals were visualized by predicting the centroids of each PC axis and plotting these as ordinations, comparing observed and predicted centroids for each plant species. These centroid residuals were then tested for phylogenetic signal using Blomberg’s K (*phytools* package, n=999 permutations). All residuals except for PCoA axis 2 for fungi (p < 0.016) were absent of a phylogenetic signal.

To assess feature importance for our models described above, we calculated Shapley Additive exPlanations (SHAP) values for each dataset and microbial group using *fastshap* [23]. SHAP analyses are a method used in machine learning to quantify the contribution of each feature to model predictions, allowing for consistent interpretation of variable influence. Recently, SHAP analyses have been used to connect host phenotypes to microbiome community composition [24, 25]. SHAP values work by estimating how much each feature contributes to shifting a model’s prediction relative to the average prediction across all observations considering all possible combinations of features. In this context, SHAP values indicate how variation in root traits shifts microbial community composition along a given PCoA axis. A SHAP value of zero means the trait has no effect relative to the mean (centered and scaled) community composition across all plant species, while positive SHAP values indicate the trait nudges community composition toward the positive end of the PCoA axis, and negative SHAP values indicate a shift toward the negative end.

We used absolute SHAP values to rank traits by their overall importance and then assessed the relationships between each trait and its SHAP values to understand how trait variation impacts microbial composition. These relationships were visualized using locally estimated scatterplot smoothing (LOESS) curves to visualize nonlinear trends between scaled trait values and their influence on microbial community structure.

*Bayesian models:*

Models were run for 5000 iterations (500 burn-ins) across four chains. Models were examined for rhat values less than or equal to one and for presence of divergent transitions. Divergent transitions occasionally occurred in the phosphorus only and greenhouse only models but were absent when considering species or phylogeny random effects. We conclude that models without species or phylogenetic information are likely weakly informative for explaining variation in rhizosphere community composition. However, because our goal was to compare models that include random effects for species and phylogeny, we considered this to have little impact on our conclusions.

Model comparison was conducted by calculating the Watanabe-Akaike Information Criterion (WAIC) and Bayesian R² for each fitted model to assess explanatory power [26]. However, overlapping WAIC scores within the standard error of each estimate prevented the identification of a single best model among those including species and phylogeny effects. Given this similarity, all models were further evaluated. To quantify the contribution of host species and phylogeny to microbiome variation, intraclass correlation coefficients (ICCs) were calculated from the posterior distributions of model parameters, following the recommendations of [Bürkner](https://cran.r-project.org/web/packages/brms/vignettes/brms_phylogenetics.html) [27, 28]. ICC measures the proportion of total variance that can be attributed to a grouping variable, in our case indicating how strongly individuals within the same group (i.e., species or phylogenetic lineage) resemble each other in microbiome composition. ICC values were extracted from models including species and/or phylogeny effects and represent the proportion of variance attributable to each grouping factor. High ICC values indicate strong clustering of microbiome composition driven by species identity or shared evolutionary history of plant hosts, while low ICC values suggest limited influence of these factors. As model comparisons did not identify a single best model based on WAIC values, mean ICC values (and standard deviations across compared models) were summarized across all models for each principal coordinate axis (Tables S2-S4). The same modeling approach for microbiome community composition was applied to root traits to identify if they are structured by host evolutionary history or species-specific adaptations.

**Supplemental Figures:**


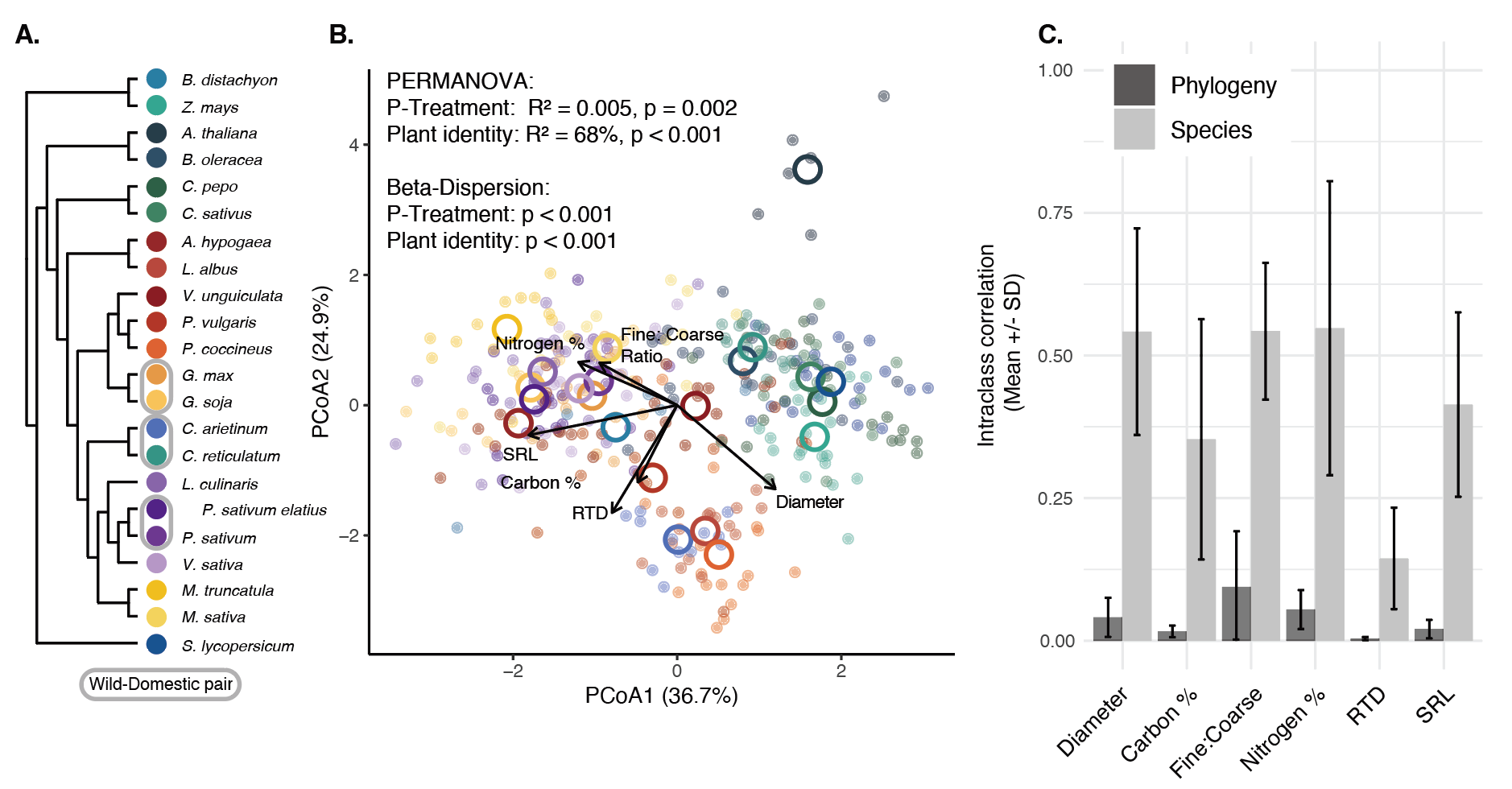


**Figure S1:** A) Phylogenetic tree of host plants, with tip colors indicating plant species and grey ellipses highlighting wild and domesticated crop pairs. (B) PCoA ordination of root traits including diameter, carbon %, the ratio of fine:coarse roots, root tissue density (RTD), and specific root length (SRL), colored by plant species. Large circles represent species centroids. PERMANOVA detected significant differences in plant identity and a significant but weak effect of phosphorus condition (P-Treatment); however, tests for homogeneity of variance indicated unequal dispersion across both factors, so these results should be interpreted with caution. (C) Variance partitioning of root traits using Bayesian models estimating species effects versus phylogenetic signal, expressed as intraclass correlation coefficients (ICCs). Across all traits, species effects exceeded phylogenetic effects.


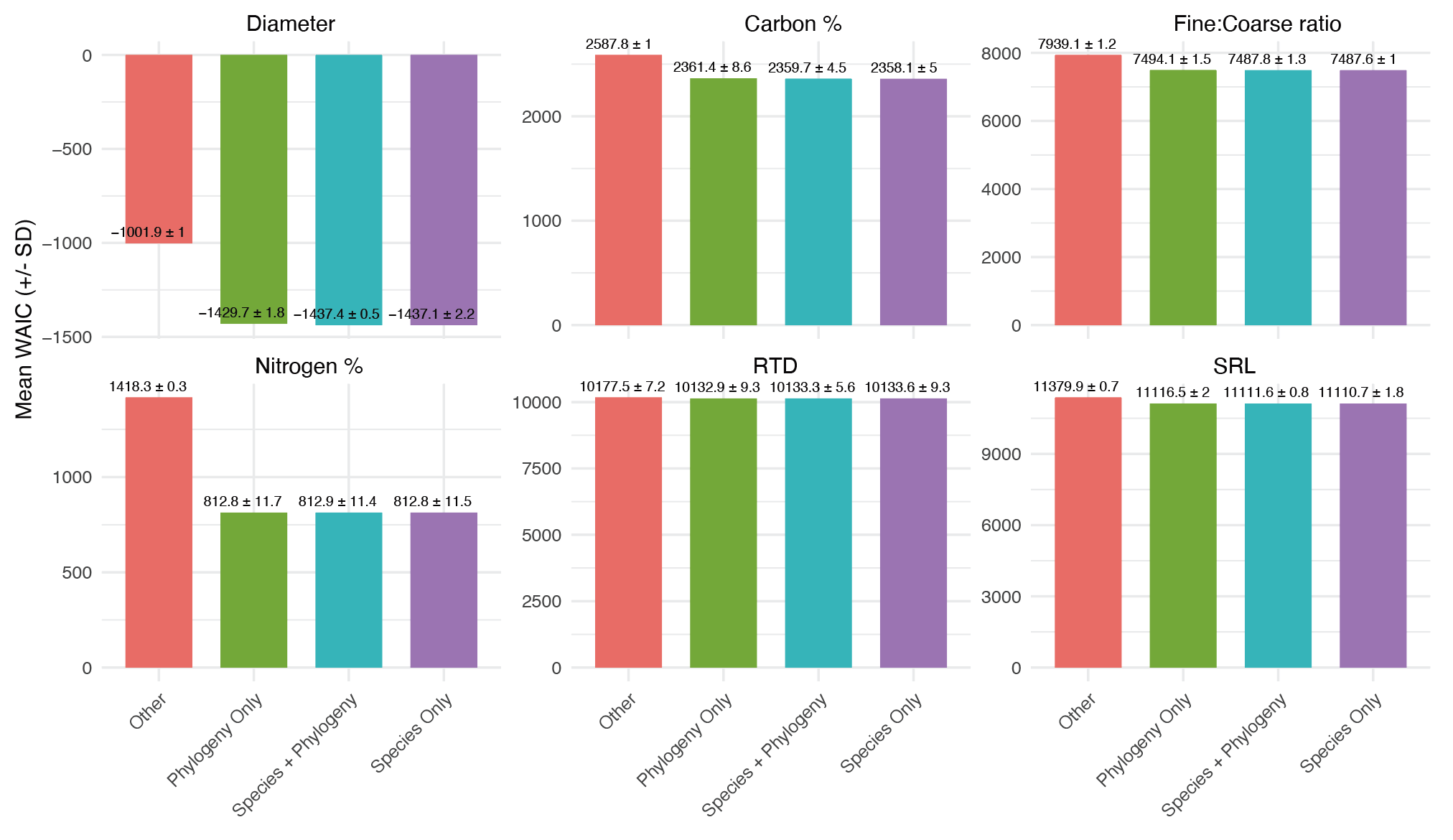


**Figure S2: WAIC values across all model versions for each root trait.** Each facet represents a different root trait, with bars showing the mean WAIC values ± standard deviation. Model versions are grouped by type and colored accordingly: red for “other” models with only greenhouse block or phosphorus conditions, green for phylogeny random effects, blue for combined species and phylogeny random effects, and purple for species random effects. Across all traits, models including species, phylogeny, or both random effects consistently performed best, compared to the models without species or phylogeny information.


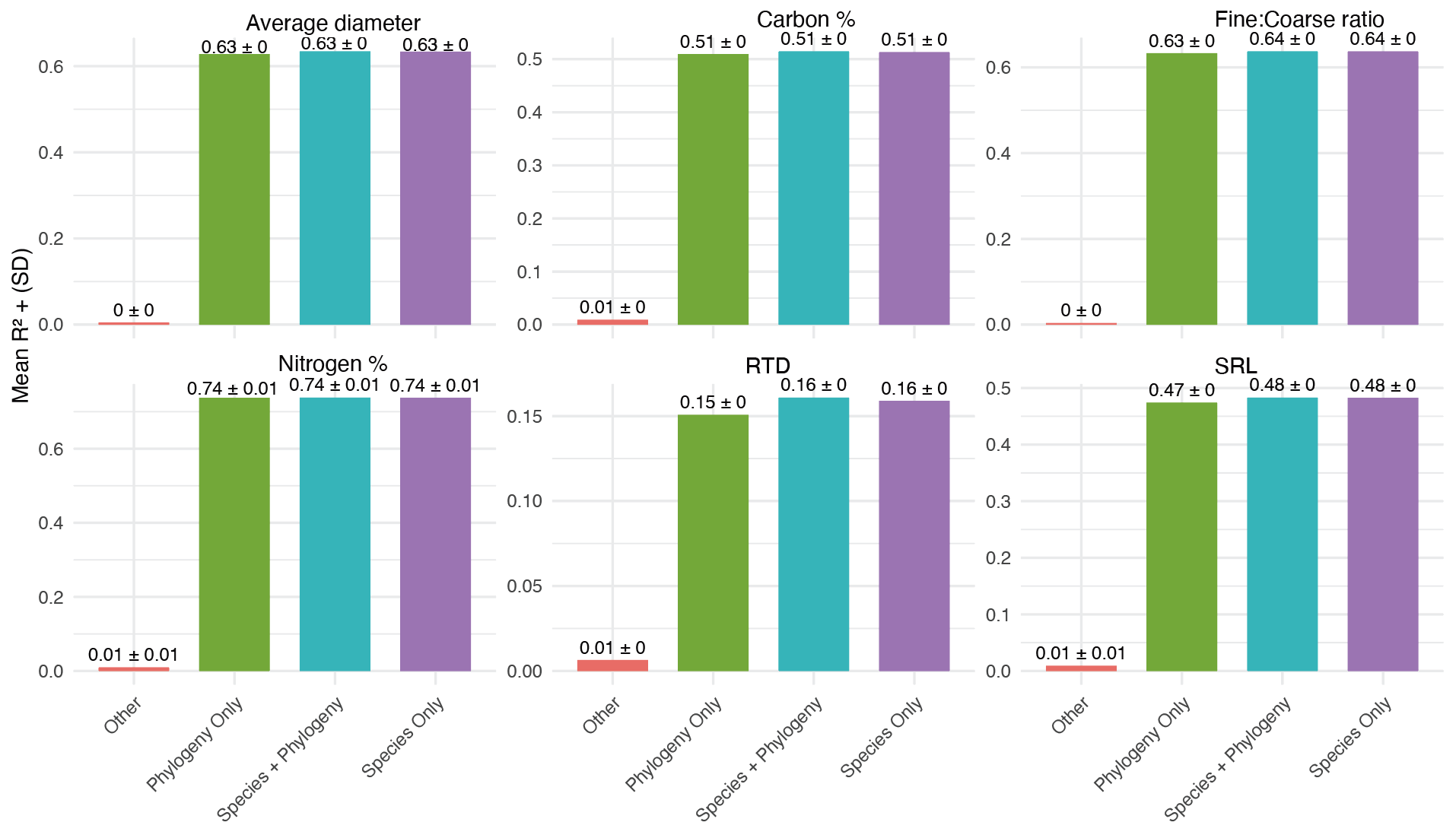


**Figure S3: Bayesian R² values across all model versions for each root trait**. Each facet represents a different root trait, with bars showing the mean R² ± standard deviation. Model versions are grouped by type and colored accordingly: red for “other” models with only greenhouse block or phosphorus conditions, green for phylogeny random effects, blue for combined species and phylogeny random effects, and purple for species random effects. Across all traits, models including species, phylogeny, or both random effects consistently performed best, explaining substantially more variance than models with phosphorus conditions and/or greenhouse block.


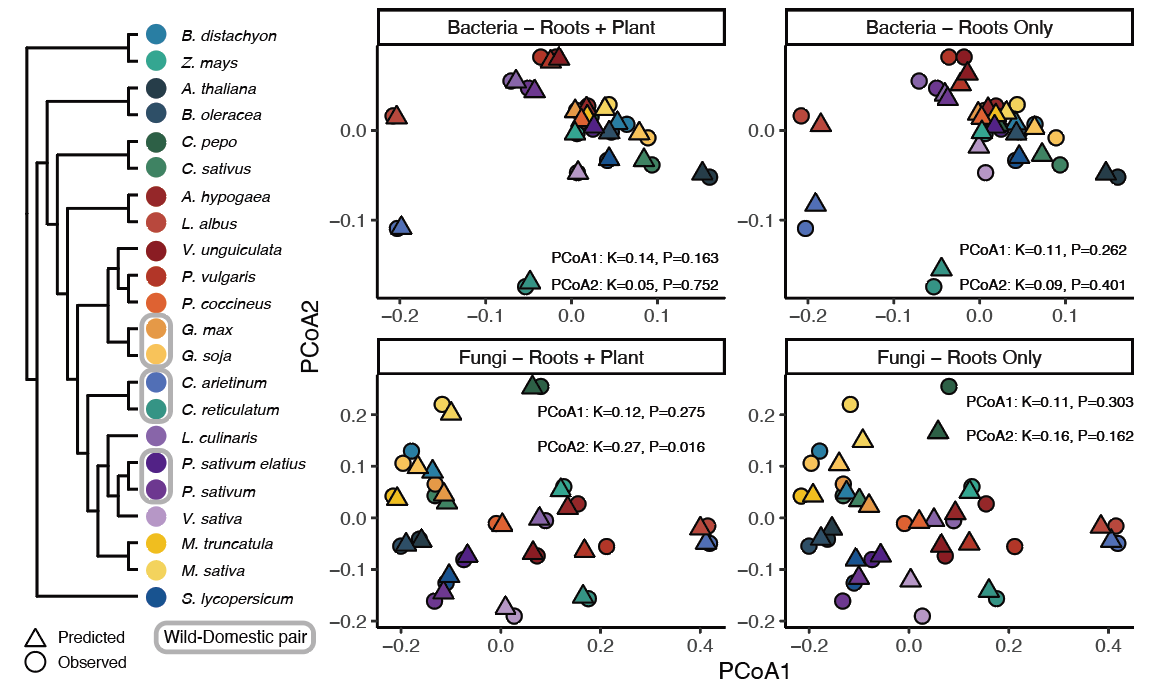


**Figure S4: Visualization of microbiome community centroids and phylogenetic relationships.** The left panel shows the plant phylogeny with tip colors corresponding to plant species centroids in ordination space. Right panels display observed (circles) and predicted (triangles) PC centroids for each plant species, based on Random Forest models of microbial community composition. Variance explained by each PC axis is reported in the main text for each model. Facet titles indicate the model type, and each facet includes results from a phylogenetic signal test on model residuals using Blomberg's K with 999 permutations. No significant phylogenetic signal was detected in residuals, except for Fungi - Roots + Plant on PC2.


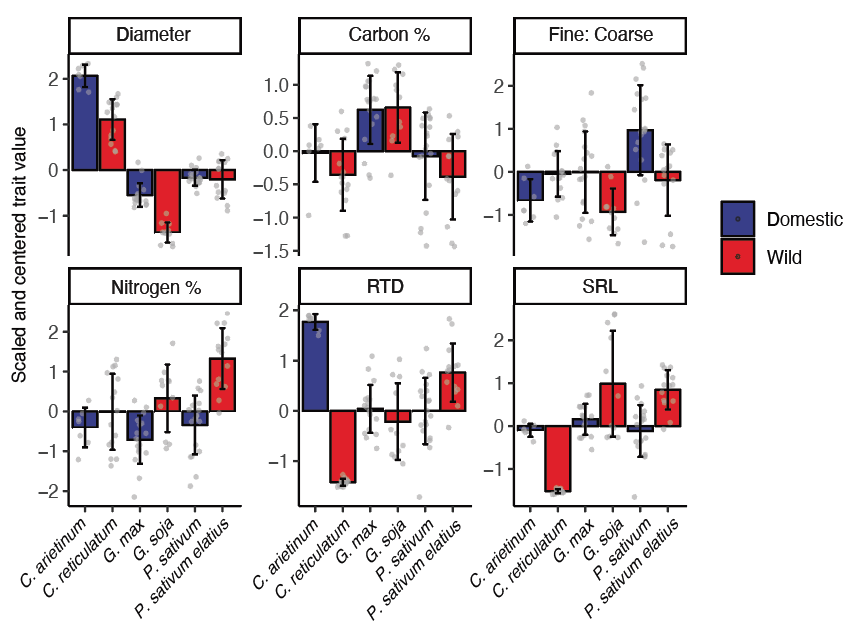


**Figure S5. Comparison of root traits in crops and their wild relatives:** Each panel displays a different root trait: root diameter, carbon percentage, the ratio of fine to coarse roots, nitrogen percentage, root tissue density (RTD), and specific root length (SRL). Bars represent the mean, and error bars indicate standard deviation. Individual data points are shown as dots. Red indicates wild relatives, while blue represents domesticated species. The x-axis lists species, including three pairs of crops and wild relatives (crop = *C. arietinum*, wild relative = *C. reticulatu*m), (crop = *G. max*, wild relative = *G. soja*), (crop = *P. sativum*, wild relative = *P. sativum elatius*).

**Figure S6: Rarefaction curve for bacterial richness (Chao1 index).** Each curve represents a different plant species, and all curves reach an asymptote, indicating that sequencing depth was sufficient to capture the majority of bacterial diversity present in the samples.

**Figure S7: Rarefaction curve for fungal richness (Chao1 index).** Each curve represents a different plant species, and nearly all curves reach an asymptote, indicating that sequencing depth was sufficient to capture the majority of fungal diversity present in the most samples. However, a few samples did not reach an asymptote.

**Figure S8.** **Principal coordinates analysis (PCoA) of bacterial and fungal community composition based on Bray–Curtis dissimilarity.** Control pots without plants are shown in blue, while study pots grown with plants are shown in red. Control pots received the same nutrient additions and watering regime as planted pots. Strong clustering of samples indicates relatively homogeneous microbiome communities for both bacteria and fungi under these conditions.


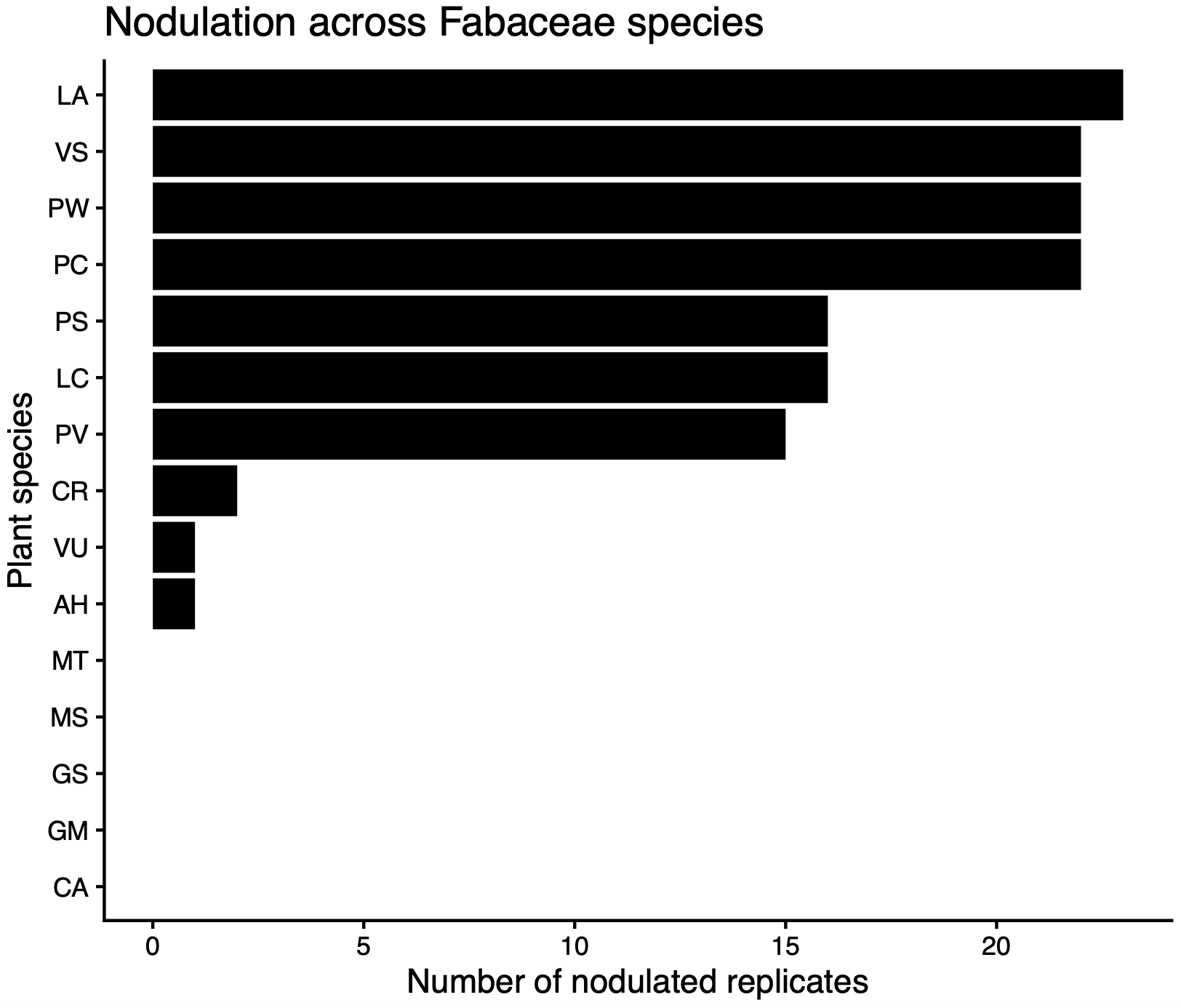


**Figure S9.** **Replicates exhibiting nodulation across plant species.** Nodulation was observed across multiple species, with high frequencies in several but not all legume species. However, symbiont identity cannot be resolved from 16S amplicon data, representing a limitation of the current approach and an important direction for future work.


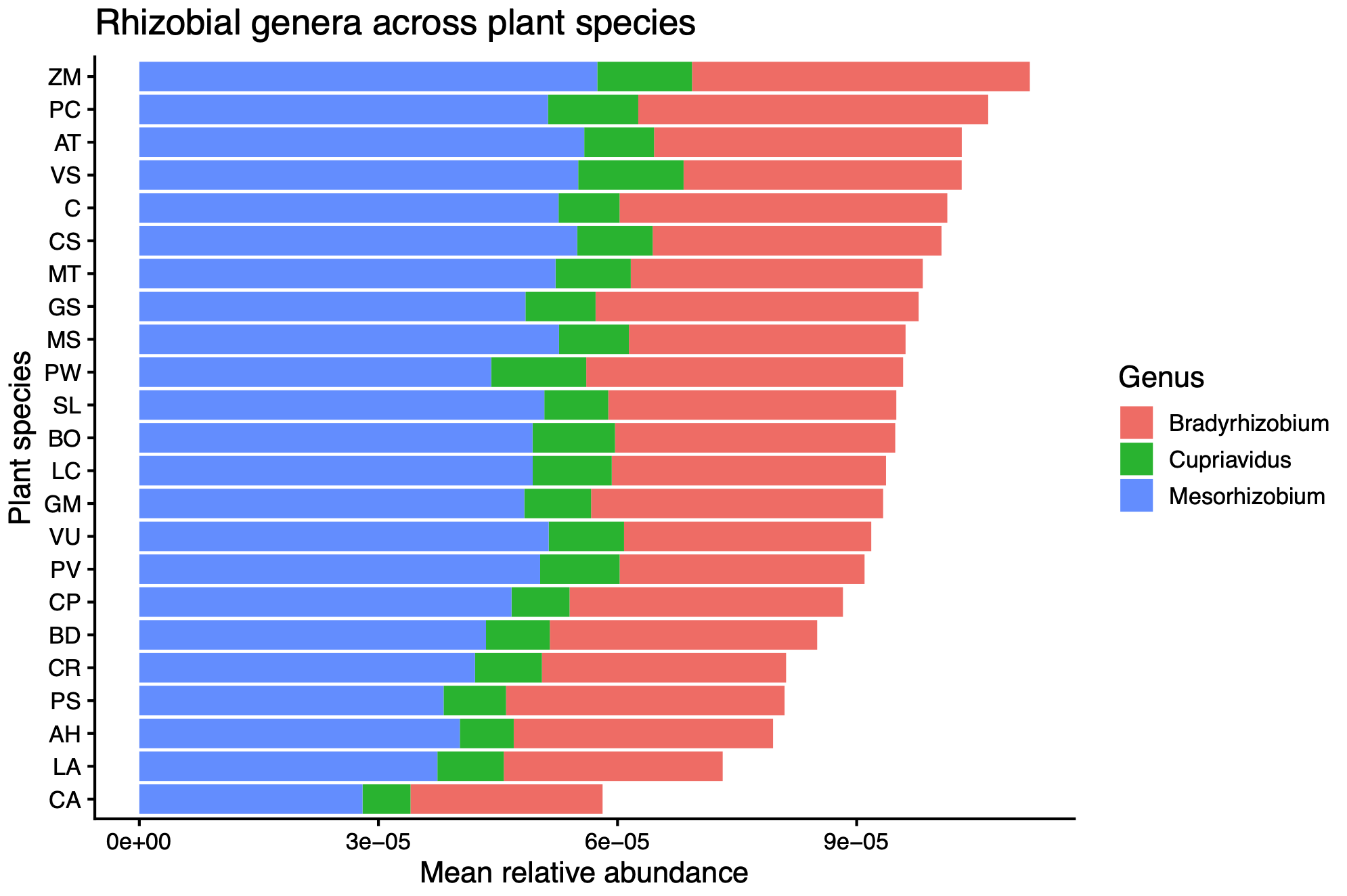


**Figure S10. Mean relative abundance of rhizobial taxa across plant species, at the genus level.** Bars show the mean relative abundance of dominant rhizobial genera to the total bacterial community within each host species. Taxonomic resolution is largely limited to the genus level using our 16s sequencing, preventing reliable identification of symbiotic partners at finer scales such as strain level.

**Table S1**: List of seeds used for plants grown in this study with their genotype and cultivar.

| **Species** | **Genotype, cultivar** |
| --- | --- |
| *Arachis hypogea* | unknown |
| *Cicer arietinum* | Flip 84-92C |
| *Cicer reticulatum* | W6 10162 |
| *Glycine max* | Williams 82 |
| *Glycine soja* | GD 50388-2 |
| *Lens culinaris* | Groene Dupuis |
| *Lupinus albus* | Amiga/Energy |
| *Medicago sativa* | unknown |
| *Medicago truncatula* | Jemalong A17 |
| *Phaseolus coccineus* | Crusader |
| *Phaseolus vulgaris* | BAT93 |
| *Pisum sativum* | Cameor |
| *Pisum sativum elatius* | unknown |
| *Vicia sativa* | Ebena |
| *Vigna unguiculata* | IT97K-499-35 |
| *Arabidopsis thaliana* | Col-0 |
| *Brassica oleracea* | Rivera |
| *Brachypodium distachyon* | GRA 787 |
| *Zea mays* | B73 |
| *Cucurbita pepo* | Orange Summer |
| *Cucumis sativus* | Tyria F1 |
| *Solanum lycopersicum* | Moneyberg |

**Table S2: File containing model comparison information for Bayesian mixed-effects models for bacterial rhizosphere community composition.**

**Table S3: File containing model comparison information for Bayesian mixed-effects models for fungal rhizosphere community composition.**

**Table S4: . Intraclass correlation coefficients (ICC ± SD) from Bayesian mixed-effects models showing the proportion of variance in root trait values explained by species identity and phylogeny. Across all traits, species identity consistently explained more variance than phylogenetic relatedness of the host plant.**

| Root trait | Species ICC ± SD | Phylogeny ICC ± SD |
| --- | --- | --- |
| Nitrogen content (%) | 0.55 ± 0.26 | 0.05 ± 0.03 |
| Fine:coarse root ratio | 0.54 ± 0.12 | 0.09 ± 0.10 |
| Average root diameter | 0.54 ± 0.18 | 0.04 ± 0.03 |
| Specific root length (SRL) | 0.41 ± 0.16 | 0.02 ± 0.01 |
| Root carbon (%) | 0.35 ± 0.21 | 0.02 ± 0.01 |
| Root tissue density (RTD) | 0.14 ± 0.09 | 0.01 ± 0.01 |

**Table S5: ANOVA tables for linear mixed-effects models of scaled root traits. Model predictors are fixed effects of domestication status, genus (pair), their interaction and phosphorus treatment. Greenhouse block was included as a random effect for all models.**

| **Trait** | **Effect** | **Sum.Sq** | **Mean.Sq** | **NumDF** | **F Value** | **P Value** |
| --- | --- | --- | --- | --- | --- | --- |
| SRL | Domestication_status | 0.3778 | 0.3778 | 1 | 1.1415 | 0.2888 |
| SRL | Pair | 23.6586 | 11.8293 | 2 | 35.7428 | < 0.0001 |
| SRL | Phosphorus | 0.4271 | 0.4271 | 1 | 1.2906 | 0.2598 |
| SRL | Domestication_status:Pair | 18.8887 | 9.4443 | 2 | 28.5366 | < 0.0001 |
| RTD | Domestication_status | 14.7522 | 14.7522 | 1 | 51.9434 | < 0.0001 |
| RTD | Pair | 3.0674 | 1.5337 | 2 | 5.4003 | 0.0065 |
| RTD | Phosphorus | 0.1964 | 0.1964 | 1 | 0.6917 | 0.4084 |
| RTD | Domestication_status:Pair | 47.7132 | 23.8566 | 2 | 84.0006 | < 0.0001 |
| Avg_Diameter | Domestication_status | 6.8035 | 6.8035 | 1 | 70.5442 | < 0.0001 |
| Avg_Diameter | Pair | 73.7636 | 36.8818 | 2 | 382.4218 | < 0.0001 |
| Avg_Diameter | Phosphorus | 0.1602 | 0.1602 | 1 | 1.6608 | 0.2014 |
| Avg_Diameter | Domestication_status:Pair | 3.3897 | 1.6949 | 2 | 17.5738 | < 0.0001 |
| N__root_percent | Domestication_status | 16.8011 | 16.8011 | 1 | 34.0312 | < 0.0001 |
| N__root_percent | Pair | 8.0722 | 4.0361 | 2 | 8.1753 | 0.0006 |
| N__root_percent | Phosphorus | 1.7078 | 1.7078 | 1 | 3.4592 | 0.0672 |
| N__root_percent | Domestication_status:Pair | 5.3743 | 2.6871 | 2 | 5.4429 | 0.0064 |
| Fine_to_Coarse | Domestication_status | 4.3746 | 4.3746 | 1 | 6.4984 | 0.0128 |
| Fine_to_Coarse | Pair | 12.2655 | 6.1328 | 2 | 9.1101 | 0.0003 |
| Fine_to_Coarse | Phosphorus | 0.2567 | 0.2567 | 1 | 0.3813 | 0.5389 |
| Fine_to_Coarse | Domestication_status:Pair | 10.3003 | 5.1501 | 2 | 7.6505 | 0.001 |
| C_root_percent | Domestication_status | 2.6303 | 2.6303 | 1 | 4.1761 | 0.0447 |
| C_root_percent | Pair | 19.6385 | 9.8193 | 2 | 15.5902 | < 0.0001 |
| C_root_percent | Phosphorus | 2.7714 | 2.7714 | 1 | 4.4002 | 0.0397 |
| C_root_percent | Domestication_status:Pair | 3.0911 | 1.5456 | 2 | 2.4539 | 0.0936 |

**References:**

1. Jin Y, Qian H. V.PhyloMaker2: An updated and enlarged R package that can generate very large phylogenies for vascular plants. *Plant Divers* 2022;44:335–339. https://doi.org/10.1016/J.PLD.2022.05.005

2. Rognes T et al. VSEARCH: a versatile open source tool for metagenomics. *PeerJ* 2016;4. https://doi.org/10.7717/PEERJ.2584

3. Özkurt E et al. LotuS2: an ultrafast and highly accurate tool for amplicon sequencing analysis. *Microbiome* 2022;10:1–14. https://doi.org/10.1186/S40168-022-01365-1

4. Hauswedell H, Singer J, Reinert K. Lambda: The local aligner for massive biological data. *Bioinformatics* 2014;30. https://doi.org/10.1093/BIOINFORMATICS/BTU439

5. Bengtsson-Palme J et al. Improved software detection and extraction of ITS1 and ITS2 from ribosomal ITS sequences of fungi and other eukaryotes for analysis of environmental sequencing data. *Methods Ecol Evol* 2013;4:914–919. https://doi.org/10.1111/2041-210X.12073

6. Callahan BJ et al. DADA2: High-resolution sample inference from Illumina amplicon data. *Nat Methods* 2016;13:581–583. https://doi.org/10.1038/nmeth.3869

7. Bengtsson-Palme J et al. Improved software detection and extraction of ITS1 and ITS2 from ribosomal ITS sequences of fungi and other eukaryotes for analysis of environmental sequencing data. *Methods Ecol Evol* 2013;4:914–919. https://doi.org/10.1111/2041-210X.12073

8. UNITE. 2025. https://unite.ut.ee/.

9. Kõljalg U et al. Towards a unified paradigm for sequence-based identification of fungi. *Mol Ecol* 2013;22:5271–5277. https://doi.org/10.1111/MEC.12481/

10. Quast C et al. The SILVA ribosomal RNA gene database project: improved data processing and web-based tools. *Nucleic Acids Res* 2013;41:D590-6. https://doi.org/10.1093/nar/gks1219

11. McMurdie PJ, Holmes S. phyloseq: An R Package for Reproducible Interactive Analysis and Graphics of Microbiome Census Data. *PLoS One* 2013;8:e61217. https://doi.org/10.1371/JOURNAL.PONE.0061217

12. Davis NM et al. Simple statistical identification and removal of contaminant sequences in marker-gene and metagenomics data. 2017. https://doi.org/10.1101/221499

13. Reich PB. The world-wide ‘fast–slow’ plant economics spectrum: a traits manifesto. *Journal of Ecology* 2014;102:275–301. https://doi.org/10.1111/1365-2745.12211

14. Bergmann J et al. The fungal collaboration gradient dominates the root economics space in plants. *Sci Adv* 2020; 6. https://doi.org/10.1126

15. Freschet GT et al. A starting guide to root ecology: strengthening ecological concepts and standardising root classification, sampling, processing and trait measurements. *New Phytologist* 2021;232:973–1122. https://doi.org/10.1111/NPH.17572

16. Fox J, Weisberg S, Price B. Companion to Applied Regression [R package car version 3.1-3]. *CRAN: Contributed Packages* 2024. https://doi.org/10.32614/CRAN.PACKAGE.CAR

17. Yue H et al. Plant domestication shapes rhizosphere microbiome assembly and metabolic functions. *Microbiome* 2023;11:1–19. https://doi.org/10.1186/S40168-023-01513-1

18. da Costa PB et al. Soil Origin and Plant Genotype Modulate Switchgrass Aboveground Productivity and Root Microbiome Assembly. *mBio* 2022;13. https://doi.org/10.1128/MBIO.00079-22

19. Guo J et al. Seed-borne, endospheric and rhizospheric core microbiota as predictors of plant functional traits across rice cultivars are dominated by deterministic processes. *New Phytologist* 2021;230:2047–2060. https://doi.org/10.1111/NPH.17297

20. Namkung J. Machine learning methods for microbiome studies. *Journal of Microbiology* 2020;58:206–216. https://doi.org/10.1007/S12275-020-0066-8

21. Kuhn M. caret Package. *J Stat Softw* 2008.

22. Wright MN. A Fast Implementation of Random Forests [R package ranger version 0.17.0]. *CRAN: Contributed Packages* 2024. https://doi.org/10.32614/CRAN.PACKAGE.RANGER

23. Jethani N et al. FastSHAP: Real-Time Shapley Value Estimation.

24. Yan Y et al. Interpretable machine learning framework reveals microbiome features of oral disease. *Microbiol Res* 2022;265:127198. https://doi.org/10.1016/J.MICRES.2022.127198

25. Carrieri AP et al. Explainable AI reveals changes in skin microbiome composition linked to phenotypic differences. *Scientific Reports 2021 11:1* 2021;11:1–18. https://doi.org/10.1038/s41598-021-83922-6

26. Gelman A et al. R-squared for Bayesian regression models *. 2018.

27. Estimating Phylogenetic Multilevel Models with brms. https://cran.r-project.org/web/packages/brms/vignettes/brms_phylogenetics.html.

28. Barrow LN et al. Deeply conserved susceptibility in a multi-host, multi-parasite system. *Ecol Lett* 2019;22:987–998.
